# MONTREAL COGNITIVE ASSESSMENT SURVEY ADAPTATION IN A COMMUNITY-BASED OLDER ADULT SAMPLE, 2019–2020 NHANES

**DOI:** 10.1093/geroni/igae098.2611

**Published:** 2024-12-31

**Authors:** Jenny Walker, Roshni Patel, John Omura, Akilah Ali, Benjamin Olivari, Lisa McGuire

**Affiliations:** Centers for Disease Control and Prevention, Atlanta, Georgia, United States; Cyberdata Technologies (CDC Contractor), Atlanta, Georgia, United States; Centers for Disease Control and Prevention, Atlanta, Georgia, United States; Centers for Disease Control and Prevention, Atlanta, Georgia, United States; Centers for Disease Control and Prevention, Atlanta, Georgia, United States; GSA, Atlanta, Georgia, United States

## Abstract

The U.S. population is aging. Some degree of cognitive change is expected as a natural part of healthy aging. Understanding the prevalence of potential impairments in community-based samples is necessary for prioritization of public health efforts. We analyzed Montreal Cognitive Assessment Survey Adaptation (MoCA-SA) data from the 2019-2020 National Health and Nutrition Examination Survey (NHANES). The full MoCA-SA was administered to a convenience sample of 1,123 individuals aged ≥60 years. Excluding incomplete inventories yielded a final analytic sample of 895. The MoCA-SA contains 20 items and is scored 0-20, with values < 17 indicative of lower cognitive performance. Scores were also analyzed for 8 subscales: orientation, naming, visual construction, executive function, attention, language fluency, abstraction, and memory. Estimates were stratified across three age categories: 60-69, 70-79, and ≥80 years. Overall, 71.2% (unweighted; 95% CI 68.2-74.1) of participants aged ≥60 years scored < 17. People aged ≥80 years scored significantly lower on the orientation and memory and higher on abstraction than those aged 60-69 years. No age-related differences were identified in the remaining subscales. While changes in cognition are a normal part of aging, not all changes are healthy. Only a healthcare provider can diagnose cognitive impairments such as Alzheimer’s disease. People experiencing changes in cognition that interfere with daily life should speak with their healthcare provider about their concerns and ways to promote brain health. Additional work is needed to determine how the MoCA-SA corresponds to cognitive functioning in daily life and which subscales may drive differences in community samples.

